# [Corrigendum] Long non-coding RNA CCAT2 functions as an oncogene in hepatocellular carcinoma, regulating cellular proliferation, migration and apoptosis

**DOI:** 10.3892/ol.2026.15832

**Published:** 2026-08-26

**Authors:** Ning Zhou, Zhongzhou Si, Ting Li, Guangshun Chen, Zhongqiang Zhang, Haizhi Qi

Oncol Lett 12: 132–138, 2016; DOI: 10.3892/ol.2016.4580

Subsequently to the publication of the above paper, an interested reader drew the authors' attention to the fact that, regarding the cell migration assay experiments shown in Fig. 4A on p. 136, the “Negative control” data panels for the HepG2 and the HuH7 cell lines contained an overlapping section of data, suggesting that these data had been incorporated into this figure incorrectly.

Upon investigating their original data, the authors have realized that the microscopic image representing the negative control group for the HuH7 cell line was included in Fig. 4A in error. The data in this panel have been replaced with correct data for the negative control group for the HuH7 cell line, and the accompanying statistical analyses have been rectified accordingly. The revised version of Fig. 4 is shown on the next page. It is important to note that the replacement of the data in this figure has not significantly changed either the results or the conclusions reported in this study.

All the authors agree with the publication of this Corrigendum, and are grateful to the Editor of *Oncology Letters* for allowing them the opportunity to publish this Corrigendum; moreover, they apologize to the readership for any inconvenience caused.

## Figures and Tables

**Figure 4.**
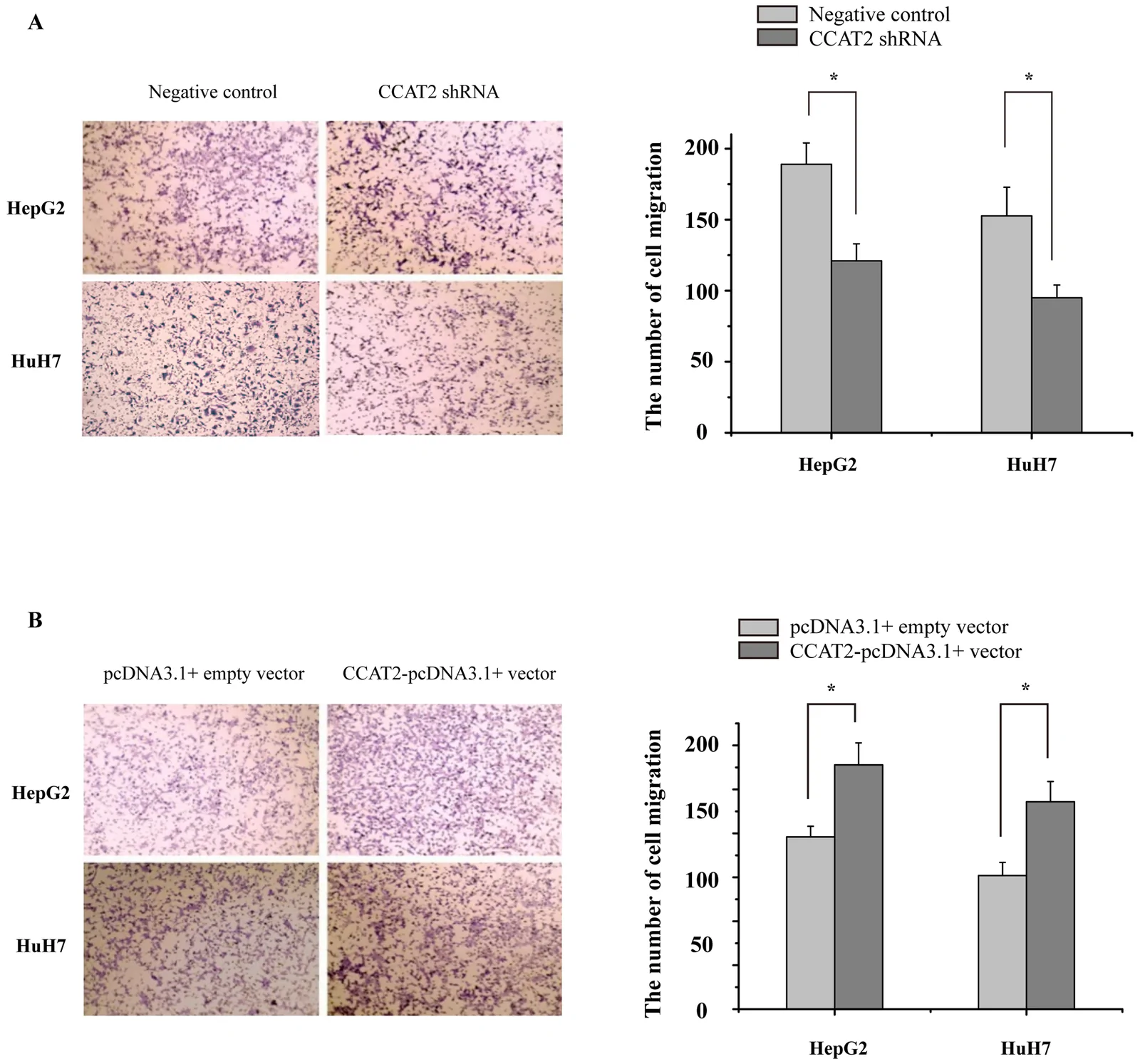
CCAT2 promotes HCC cell migration. (A) HepG2 and HuH7 cells transfected with CCAT2 shRNA or negative control. The suppression of CCAT2 expression significantly reduced the migration rate of the HCC cell lines when compared to the control. (B) HepG2 and HuH7 cells transfected with CCAT2-pcDNA3.1(+) vector or pcDNA3.1(+) empty vector. Overexpression of CCAT2 significantly increased the migration rate of the HCC cell lines when compared to the control. Values are expressed as the mean ± standard deviation. Each experiment was repeated three times. *P<0.05.

